# Causal mediation approaches for understanding pathways to inequalities and policy entry points: examples from early years health and development

**DOI:** 10.1136/jech-2025-224260

**Published:** 2025-11-03

**Authors:** Anna Pearce, Steven Hope, Michael J Green, John W Lynch, Joost Oude Groeniger, Bianca De Stavola, Russell M Viner, Daniela K Schlüter, David Taylor-Robinson

**Affiliations:** 1 MRC/CSO Social and Public Health Sciences Unit, Institute of Health and Wellbeing, University of Glasgow, Glasgow, UK; 2 Mohn Centre for Children's Health and Wellbeing, School of Public Health, Imperial College London, London, England, UK; 3 Department of Obstetrics and Gynecology, Duke University School of Medicine, Durham, North Carolina, USA; 4 Better Start, The University of Adelaide, Adelaide, South Australia, Australia; 5 Department of Public Health, Erasmus MC University Medical Center, Rotterdam, Netherlands; 6 UCL Great Ormond Street Institute of Child Health, London, UK; 7 Department of Public Health, Policy and Systems, Institute of Population Health, University of Liverpool, Liverpool, UK

**Keywords:** METHODS, POLICY, COHORT STUDIES, Life course epidemiology, LONGITUDINAL STUDIES

## Abstract

The reduction of health inequalities has been a priority of researchers, decision-makers and practitioners for many years. Advances in causal mediation analysis offer great promise for identifying intervention targets and inferring how policy actions might alter health inequalities. However, these methods are sometimes presented in a manner that is not accessible to the wider community of health researchers. Causal mediation methods also have a range of limitations and assumptions that have implications for their application and the interpretation of results. In this paper, we consider three types of questions that can be used to guide policy actions to reduce health inequalities, addressed using causal mediation methods: (1) which mediating pathways offer most promise for the reduction of health inequalities and should be the focus of further, more indepth analysis? 2) In the face of two competing pathways, which one is most likely to lead to a narrowing of health inequalities? 3) What would be the impact of a hypothetical intervention on one specific mediating pathway when implemented under different scenarios? Focusing on early years’ health, we use real life examples of the application of causal mediation methods to address these three types of question. In doing so, we discuss the relative strengths and limitations of these methods and introduce key mediation concepts relevant to health inequalities researchers.

**WHAT IS ALREADY KNOWN ON THIS TOPIC**

- Causal mediation methods are gaining popularity in the field of health inequalities research, as a means to identifying which causal pathways might be the most fruitful points for action and the potential impacts of different modes of implementation. However, there is a need for a more accessible description of causal mediation methods.

**WHAT THIS STUDY ADDS**

- Using published examples of the application of causal mediation methods to policy-relevant health inequalities questions, we showcase the potential of these methods and some of the important pitfalls to consider. While acknowledging that the field continues to develop, this article describes key concepts and applications and signposts further reading.

**HOW THIS STUDY MIGHT AFFECT RESEARCH, PRACTICE OR POLICY**

- This theory and methods review supports researchers to know when, when not and how to use causal mediation methods, leading to higher quality, solution-focused health inequalities research.

## Background

 Socioeconomic inequalities in child health and well-being persist, despite being a public health priority. But how to reduce these inequalities? An obvious place to start is the root cause, improving the socioeconomic circumstances that create social inequality. A complementary approach is to identify and intervene on risk factors that lie on the causal pathway between socioeconomic circumstances and child health.^1^,^4^

We can investigate the extent to which the impact of socioeconomic circumstances (exposure) on child health (outcome) is explained by higher prevalence of risk factors (mediators) in disadvantaged compared with more advantaged groups. Unpicking these causal pathways allows policymakers to understand the relative benefits of intervening on upstream causes, such as poverty, versus risk factors on the causal pathway from poverty to poor health outcomes (eg, breastfeeding behaviour, parental mental health).

Large, double-blind, randomised control trials with well-defined exposures and no informative loss-to-follow-up are considered gold standard designs for estimating causal effects. However, many policies that might be expected to reduce health inequalities cannot be randomised because this would be neither ethical nor practical. Well-designed natural experiments offer an alternative but can only be used to evaluate the impacts of policies that have already been enacted. Thus, it is common for policies intended to improve health to be enacted without their effectiveness having been assessed (eg, the Troubled Families programme^5^ or the roll-out of Sure Start Children’s centres^6^ in the UK).

Epidemiologists have attempted to disentangle pathways linking socioeconomic circumstances to later health using mediation analysis of observational data.^7^,^9^ Sewall Wright’s development of path analysis in the ‘30s was a precursor to directed acyclic graphs (DAGs), now widely used in epidemiology to visually represent assumed causal pathways.^10^ Regression models have been widely used to examine the association between socioeconomic circumstances and outcomes, with adjustment for variables representing ‘explanatory pathways’.^7 9 11 12^ The degree of attenuation after this adjustment was taken to indicate the extent of mediation. However, this approach may produce misleading results for several reasons, including non-collapsibility of odds ratios (ORs)^13^ and unmet assumptions about the absence of confounding of the mediator-outcome relationship, especially when confounding factors are downstream of the exposure; that is, they are mediators themselves (see Exposure-induced mediator-outcome confounding, table 1; traditional multivariable regression, box 1).

**Table 1:** Glossary – mediation concepts in the context of health inequalities (listed alphabetically)

Concept	Description	Practical example relating to health inequalities	Additional notes, including assumptions*
Baseline confounder	A common cause of the exposure and the outcome.	Young age at first live birth can affect maternal academic qualifications and children’s health and is therefore a baseline confounder of the relationship between maternal academic qualifications and child health.	
Controlled direct effect	The effect of the exposure on the outcome when the mediator is held at a given level.	The controlled direct effect of low income on health not acting via self-regulation is given by the effects of low income if everyone were poor versus if everyone were not poor when, for example, self-regulation was held at the mean level observed in the study population.	The controlled direct effect (CDE) relies on fewer assumptions than *natural effects*; specifically, it is possible to remove exposure-induced mediator-outcome confounding by setting the mediator to a specific level.
Counterfactual outcome	In the context of an exposure-outcome relationship, this is the outcome which would have occurred if an alternative exposure (to that observed) had been experienced.	For a child living in poverty, there is one counterfactual outcome: the health outcome they would have had if they had instead not experienced poverty.	In the case of a binary exposure (eg, poverty status) there is only one counterfactual outcome. Where exposures have multiple values (eg, income), there are many counterfactual outcomes, although not all may be of interest.
Direct effect	The effect of an exposure on an outcome not acting through the specified mediating pathway(s) of interest.	The effect of income on health is not acting through self-regulation. This includes any indirect effects via any mediating pathway not adjusted for in the analysis (eg, maternal mental health).	
Exposure-induced mediator-outcome confounder (also known as: intermediate confounder)	A class of mediator-outcome confounder which is affected by the exposure.	Maternal mental health may be an exposure-induced mediator outcome confounder in an analysis exploring the indirect effects of income (the exposure) on child health (the outcome) via child self-regulation (the mediator).	In the context of health inequalities research, where socioeconomic factors are the exposure, almost all mediator-outcome confounders will be ‘exposure-induced’. Where exposure-induced mediator-outcome confounding is present, it is not possible to identify natural effects (but it may still be possible to identify their interventional analogues – see below).
Exposure-mediator interaction	This occurs when the effect of the exposure depends on the value of the mediator (or vice versa). Through a health inequalities lens, this mechanism can be thought of as differential susceptibility.	If the detrimental effects of low self-regulation skills on health are greater for those living in poverty versus those not (*assuming* poverty causes lower self-regulation skills).	
Indirect effect	The effect of an exposure on an outcome acting through a given mediator.	The effect of income on children’s academic achievement acting through self-regulation skills.	
Interventional analogue direct and indirect effects	Counterfactual values of the mediator are randomly assigned, as if by intervention, to all individuals. Mediator values are drawn from the observed distribution among those with the relevant level of exposure, conditional on confounders.	To estimate interventional analogue effects of low income on health via self-regulation (indirect) and not via self-regulation (direct), new counterfactual values of the mediator (self-regulation) are assigned. For all individuals (including those living in poverty), these are randomly drawn from the distribution of observed self-regulation values among children who were not living in poverty.	Interventional analogue effects circumvent the problem of exposure-induced mediator-outcome confounding by assigning values of the mediator, as if they had been assigned by randomised intervention.
Mediator	A factor which is caused by the exposure and affects the outcome. Through a health inequalities lens, this mechanism can be thought of as differential exposure.^30^	Early life self-regulation skills are affected by income and affect child health; they may therefore mediate the relationship between income and child health.	
Mediator-outcome confounder	A factor that affects both the mediator and outcome.	Sex affects self-regulation skills and some health outcomes	
Natural direct effect	Effect of changing the exposure, with the mediator fixed at whatever (counterfactual) value it would have taken if the exposure were set at its original level	The natural direct effect of low income on academic achievement is given by the change in the risk of the outcome when the exposure is altered from its observed value (poverty) to its counterfactual value (not poverty), while the level of self-regulation is held at the observed value. Simply put, only the exposure is altered, and the mediator is kept at its observed value. Thus, we are estimating the effect of the exposure not acting through the mediator (the natural direct effect).	Relies on the cross-world independence assumption, or in other words, represents a mathematical quantity that could not be estimated in the real world. The policy relevance of natural effects has therefore been questioned.^31^
Natural indirect effect	Effect of changing the mediator between the values it would have with and without the exposure, when the exposure is present	The natural indirect effect of low income on health through self-regulation is given by: the effect on poor health if the level of self-regulation were to be altered from the observed value (when the exposure=poverty) to a counterfactual value (when exposure=not living in poverty). The exposure is held at its observed value (poverty). Thus, we are estimating the effect of the exposure acting only via the mediator (the natural indirect effect).	Relies on the cross-world independence assumption, or in other words, represents a mathematical quantity that could not be estimated in the real world. The policy relevance of natural effects has therefore been questioned.^31^
Potential outcome	In the context of an exposure-outcome relationship, this is any possible outcome that could occur under any possible value of an exposure.	For a child living in poverty, there are two potential outcomes: the health they experienced when living in poverty and the health they would have experienced if not living in poverty.	Similar to the idea of a counterfactual outcome, but potential outcomes include both the observed and all possible *counterfactual outcomes*
Total effect	The effect of an exposure on an outcome after adjustment for baseline confounding.	The effect of household income on child health after adjusting for ethnicity, family structure, maternal education and maternal age at first live birth.	Assumes no unmeasured *baseline confounding* and perfect measurement of exposure, outcome and confounders.

* Please note that many of the assumptions are not specific to these methods.

Box 1 **Methods for mediation analysis covered in the introduction and the applied examples**

This box provides a brief explanation of the methods used in the examples with accompanying references; it does not provide a comprehensive description of all relevant mediation methods.

**Traditional multivariable regression (see the Introduction section**) is a common and easily implemented approach, which consists of fitting multivariable regression models for the exposure-outcome relationship (adjusting for confounding) and then adjusting for the mediator(s) and all measured mediator-outcome confounders. The rationale behind the adjustment for the confounders is to block back-door pathways, while the adjustment for unconfounded mediator(s) is meant to indicate an indirect effect of the exposure. While the multivariable regression approach can be used to adjust for mediator-outcome confounding, it assumes that these confounders do not lie on the causal pathway between the exposure and outcome (ie, that there is no exposure-induced mediator-outcome confounding; see Table 1). This assumption is rarely met in health inequalities research since socio-economic circumstances affect almost all aspects of life and will therefore be a cause of most confounders. In a traditional multivariable regression where exposure-induced mediator-outcome confounding is thought to be present (ie, almost always), either adjusting or not adjusting for it will produce biased effect estimates. Adjusting for exposure-induced mediator-outcome confounders (to reduce confounding on the mediator-outcome pathway) will provide biased estimates because it blocks part of the direct effect. Not adjusting for exposure-induced mediator-outcome confounders will also provide biased estimates (since the indirect pathway remains confounded).

**Flexible mediation analysis using natural effects models (Q1**) is a class of counterfactual modelling to directly parameterise the path-specific effects of interest, in the presence of one or many mediators, taking into account interactions between the variables included in mediating blocks.^32 33^ The total effect of socioeconomic factors on outcomes can be decomposed into the natural direct effect and the natural indirect effect, via one or more of the mediating blocks (under the assumptions of no intermediate confounding and independence between mediating blocks). One approach involves fitting an appropriate regression model for the outcome conditioned on mediators, exposure and confounders using the original data; replicating the dataset and adding the counterfactual exposure variable to the dataset and imputing the outcome for the counterfactuals of interest.

**Effect decomposition analysis (Q2):** in the example, *natural direct* and *indirect effects* are estimated using a method that is akin to imputation for missing data; the values of the outcome and mediator under the counterfactual exposure are not observed. New values for these can be estimated (akin to imputation) using data from others who are dissimilar on the exposure while being comparable with respect to the confounders. Modelling proceeds using potential outcomes rather than observed outcomes. This produces new lines of data, referred to as ‘scenarios’ here, which can be compared in different combinations to estimate the natural direct and indirect effects.

**Randomised Interventional Analogue (Q2):** the method used in this example, similar to a *marginal structural model*, used weighting to reduce confounding from exposure-induced mediator-outcome confounding, though regression adjustment could also be used. The value of the mediator is altered, as if it had been randomly assigned to individuals, with those values being drawn from an alternative distribution of values. The *indirect effect* is estimated by comparing scenarios where the mediator is randomly drawn from (1) the distribution of observed mediator values from individuals with the *counterfactual* exposure (eg, not in poverty) to (2) from individuals with the *observed* exposure (eg, in poverty). In both scenarios, the exposure is held at its observed value (eg, in poverty). The *direct effect* is estimated by comparing the two scenarios where the exposure takes on its counterfactual value vs its observed value. In both scenarios, the mediator value is drawn from the distribution of those with the counterfactual exposure (eg, not in poverty).

**Marginal structural models (Q3**) (to estimate the *controlled direct effect*): marginal structural models are models for the *potential outcome* (see Table 1). In practice, they are similar to a traditional multivariable regression model but use a two-step weighting procedure (inverse probability of treatment weights), with the first step accounting for exposure-outcome confounding and the second step additionally accounting for mediator-outcome confounding, conditional on the exposure, that is, it aims to balance mediator-outcome confounders within levels of the exposure (so any effect of the exposure on these remains). A marginal structural model can produce a *controlled direct effect*, that is the effect of the exposure not acting via the mediator, when the mediator is held at a particular level for everyone. If the exposure and mediator interact in their effect on the outcome, the *controlled direct effect* estimate will be correct for the level that the mediator is held at (and may differ if it were held at different levels of the mediator).

Methodological developments in counterfactual-based causal inference allow a clearer formulation and identification of mediating pathways linking adverse socioeconomic circumstances to outcomes. They have also clarified the assumptions required to apportion a mediator’s contribution to the total effect (TE) of an exposure on an outcome.^14 15^ Such methods have been applied to longitudinal data to investigate mediating pathways to health inequalities in children.^316^,^18^ By making plausible changes to mediator variables (as if through intervention), it is possible to estimate the impacts of potential interventions or policy actions on health inequalities. This approach can model a variety of scenarios for the implementation of an intervention, including combinations of eligibility, effectiveness and uptake,^19 20^ and therefore compare by how much inequalities would reduce if improvements were made on mediating risk factors.

**Figure 1:**
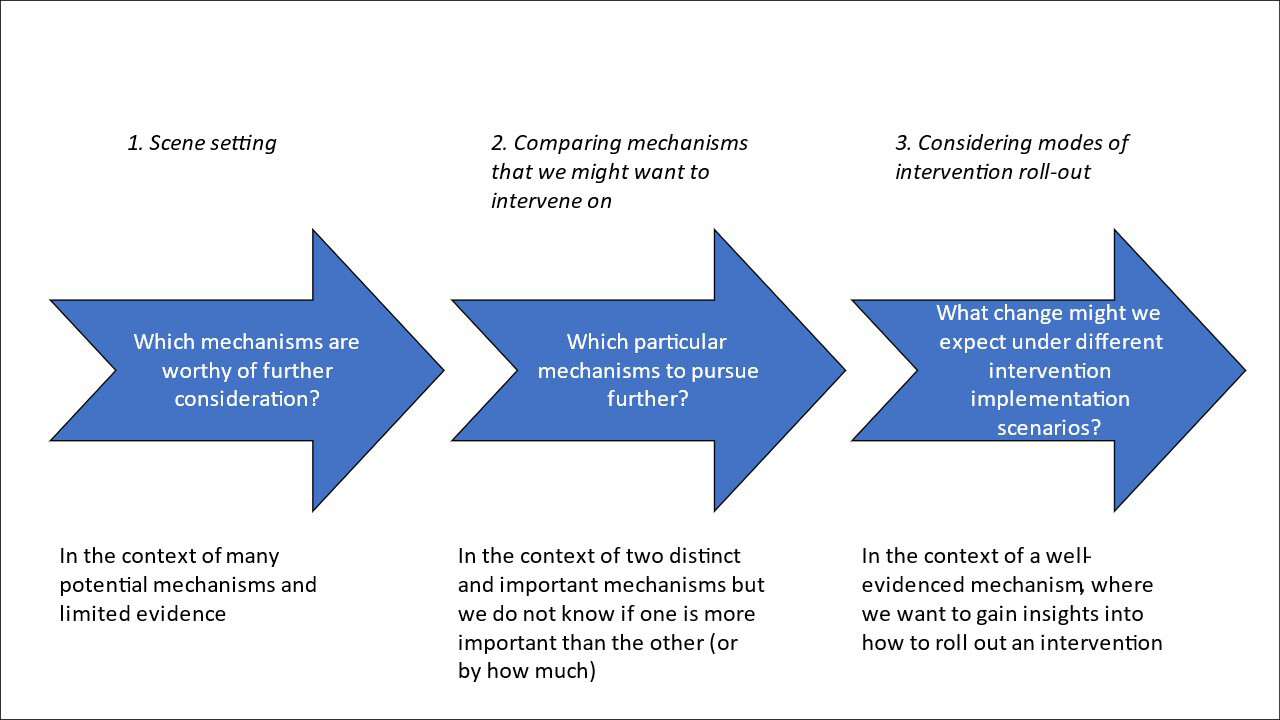
Causal mediation approaches for understanding pathways to child health inequalities and assessing promising policy options.

Here, we use three published examples to illustrate how policy-relevant questions in health inequalities research can be investigated using causal mediation approaches (figure 1). While it is not necessary to use the specific approaches outlined here, these questions and the methodological issues outlined are illustrative of how to use data to inform the selection and implementation of policy options.

Poor socioeconomic circumstances in childhood can affect health via many complex and inter-related pathways (eg, housing, nutrition, physical activity, etc). Therefore, this paper includes examples that investigate the comparative impact of multiple mediators along with the analysis of a single mediator. The first question is about scene-setting in the context of many possible mediators, leading to a hypothesis-building exercise which identifies candidate pathways worthy of consideration. The second question compares two mediating pathways where we want to know which of the two mechanisms is more important. The third question focuses on a single established pathway to estimate the potential impacts of hypothetical interventions rolled out under different implementation scenarios, such as eligibility for, or intensity of, an intervention.

In addressing these questions, we provide a simple introduction to relevant theory, including a glossary of key terms (table 1), a description of some of the current methods (box 1), the challenges and limitations of these approaches in applied health inequalities research and signposting to further reading.

### Q1: which mediating pathways look most promising for the reduction of health inequalities in the context of many possible mediators and limited evidence?

This question addresses the total extent of mediation through policy-relevant groupings of potential mediators. We are interested in exploring both the indirect effects (the extent to which socioeconomic circumstances affect the outcome via mediators) and the direct effect (the extent to which inequalities in the outcome would remain if the mediators were no longer affected by the exposure). This information can be used to guide subsequent analysis of specific pathways (see Q2–3).

#### Example

**Figure 2:**
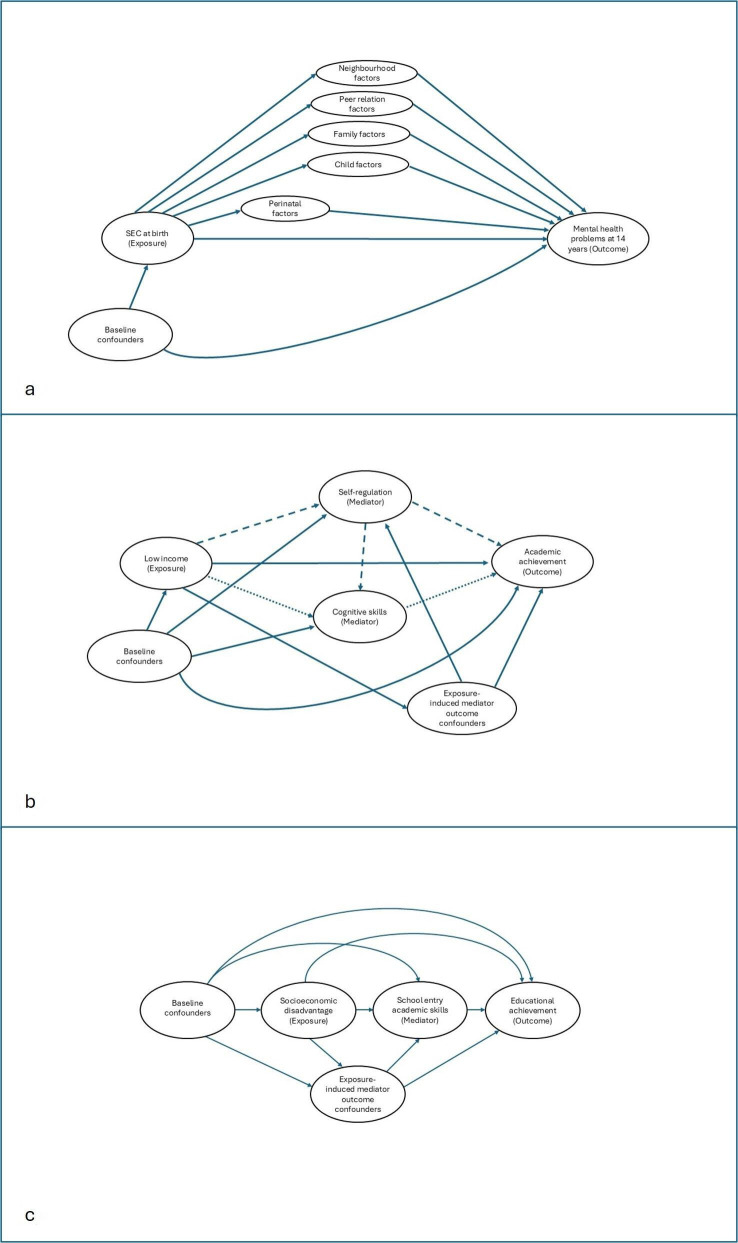
Directed acyclic graphs demonstrating (from top to bottom) the posited relationships among: (a) socioeconomic circumstances (SEC) at birth (exposure), mental health (outcome) and five ‘blocks’ of perinatal, child, family, peer-relation and neighbourhood factors (mediators); (b) low income (exposure), academic achievement (outcome) and self-regulation and cognitive skills (mediators); (c) socioeconomic disadvantage (exposure), academic achievement (outcome) and school entry academic skills (mediator).

This example used natural effects models (box 1) to estimate the TE of socioeconomic circumstances (maternal educational qualifications at birth) on adolescent mental health at age 14 (a binary measure, using the Strength and Difficulties Questionnaire) and the natural direct effect (NDE) of socioeconomic circumstances after accounting for a range of early life mediators measured up to the age of 3 years.^16^ Natural indirect effects (NIE) of socioeconomic circumstances on child mental health were estimated for meaningful ‘blocks’ of mediators (perinatal, child, family, peer-relation and neighbourhood factors), to establish whether some have greater potential than others. The DAG details the blocks of mediators (figure 2a), baseline confounders and the hypothesised lack of causal relationships between the blocks of mediators (which may not reflect reality). Interactions between mediators within each block are not shown in the DAG but are allowed for using this method.

If assumptions for these causal effects are met (see ‘Limitations’), the total causal effect of maternal education on adolescent mental health (4.40 (95% Confidence Interval (CI) 3.18, 6.07)) can be interpreted as a 4.4 times increased risk if all mothers were without qualifications vs if all mothers had qualifications. Almost two-thirds (63.9% (95% CI 50.2%, 77.6%)) of the TE was mediated by all blocks of early-life factors collectively (NIE risk ratio (RR): 1.97 (95% CI 1.63, 2.37)). Separate analyses, estimating direct and indirect effects one block at a time, assuming they act along independent pathways, showed that the block representing perinatal factors was potentially the most important, mediating around one-third of the TE.^16^

#### Limitations

Like all mediation analyses, the results can only be interpreted as causal if technical assumptions hold (no interference and consistency)^15^ in addition to conditional exchangeability assumption, which states that there is no unmeasured confounding. The method used could not adjust for exposure-induced mediator-outcome confounding, which is problematic in inequalities research (see table 1 for further explanation of this assumption and its consequences). Indeed, some mediators included in figure 2a could act as exposure-induced confounders for other mediators within a block. Nevertheless, this method has several strengths. First, it allows for an initial assessment of the joint impact of multiple pathways (given the assumption of no unmeasured confounding). The analysis allows for interactions between variables in a mediating block, thus accounting for the accumulation of risk (within these blocks).^21^ In the example shown here, the results indicated that almost two-thirds of inequalities were mediated by risk factors which may be amenable to change. Second, it provides a preliminary comparison of a range of mediators which can be helpful if the current evidence base about the relative importance of competing mediators is lacking. It is preliminary, because this method cannot accurately adjust for intermediate confounding and assumes independence between mediating blocks. In this example, the pathway comprising perinatal factors appeared most important, when compared with other pathways which were downstream in temporal ordering. These downstream pathways (including child development, peer and neighbourhood factors) are more likely to be confounded by earlier mediators. Based on the known direction of association between these mediators, the later pathways are likely to be overestimated, lending further strength to the assumption, in this example, that the perinatal pathway was most important. To unpick this further, the analysis should be extended to take into account assumptions about the sequential ordering between different blocks, using methods which are better suited to account for related mediators and intermediate confounding, which leads us to Q2.

### Q2: in the context of finite resources and an understanding of the major contributors to inequalities in a given health outcome, *where* should investment be directed or *when *in the life course?

Here, the evidence base points towards a small number of potentially important mediators (through research such as that carried out in Q1) and we want to compare their relative importance or combined contribution to understand where to focus attention. The pathways could represent distinct mediating factors (requiring different interventions) or they could be the same mediator measured at distinct time points (requiring interventions at different points of the life-course). As in Q1, the quantities we are estimating are the direct effect of socioeconomic circumstances on the outcome not via either of the two pathways, and the indirect effects of socioeconomic circumstances on the outcome via these mediating pathways, both separately and in combination. By focussing on a smaller number of pathways, we are able to adjust for measured confounding and make fairer comparisons of two mediating pathways of interest, as shown in the following example.

#### Example

This paper examined the effect of poor socioeconomic circumstances (lowest income quintile) on children’s academic achievement and compared relative mediation by preschool cognitive skills and self-regulation.^18^ As the two mediators are likely to be related, it was necessary to specify the hypothesised direction of the relationship between them. Here, the authors posited that self-regulation affects cognitive skills (figure 2b). Baseline and exposure-induced mediator-outcome confounders (see table 1 for definitions) were identified and adjusted for where possible.

The authors used a counterfactual decomposition approach to partition the total causal effect of low income into the NDE and the NIE acting via the two, related mediating pathways.^22^ The NIE is estimated for both mediators in combination and also (in two separate analyses) via: (1) the later mediator only and (2) the first mediator and its downstream paths. To avoid the assumption of no exposure-induced mediator-outcome confounding, which was not realistic, Interventional Analogues of natural effects (table 1) were also estimated, by simulating a randomised control trial of the mediators (one at a time), while adjusting for measured intermediate confounders through weighting.

Results from the Longitudinal Study of Australian Children showed around a 50% increased risk of low maths scores due to low income which is not mediated by self-regulation or cognitive ability (NDE RR: 1.46 (95% CI 1.17, 1.79)). Of the two mediators, cognitive skills had the biggest estimated contribution (NIE RR: 1.13 (95% CI 1.06, 1.22)), while the pathway via self-regulation (and its downstream impact via cognitive skills) was very small (RR: 1.05 (95% CI 1.01, 1.11)). The ‘Intervention Analogue’ (IA) approach allowed consideration of bias from *measured* confounders that could not be adjusted for in natural effects models. The estimates using these two approaches were similar.

This example indicated that inequalities in academic achievement are large (with an almost two-fold risk of low maths scores among children from less advantaged backgrounds). While collectively, cognitive skills and self-regulation contributed to around one-third of this inequality, almost three-quarters of the indirect effect was via cognitive skills and not self-regulation. The authors concluded that interventions to improve cognitive skills are likely to be more effective at reducing educational inequalities than those targeting self-regulation.

#### Limitations

A limitation of the method used in Q2 is that it is most easily operationalised using binary mediator variables. Dichotomisation can lead to the loss of important information, and therefore an underestimation of mediated effects. Measurement error can be particularly problematic when the intention is to compare mediating pathways (if one mediator is better measured than the other). As mentioned in Q1, natural effects have important limitations, including a reliance on the cross-world independence assumption, which implies an inability to address exposure-induced mediator-outcome confounding (see Exposure-induced mediator-outcome confounding and natural direct and natural indirect effects in table 1). The IA approach used in this example has the potential to overcome this assumption under certain conditions, such as perfectly measured intermediate confounders, as do controlled direct effects (CDE), referred to in the next question.

### Q3: focusing on a single mediating pathway, *what would be the impact* of a hypothetical intervention when implemented under different roll-out scenarios?

This question asks: by how much might inequalities in an outcome be reduced if a single mediator could be changed under different scenarios of intervention intensity, eligibility and uptake? Considering such scenarios is central to real-world policy decisions that have to identify the population that would benefit from the intervention while also accounting for the resources available to implement it. Causal mediation methods can be used to simulate the impact of an intervention on the mediator and to estimate the potential for an intervention to alter inequalities in an outcome under different scenarios. The quantities we are interested in estimating are the direct effects of socio-economic circumstances on the outcome under hypothetical interventions on the mediator.

#### Example

This example sought to estimate what would happen to socioeconomic inequalities in educational achievement at the age of 16 years if early school readiness were improved through effective preschool interventions (figure 2c), using data from the Avon Longitudinal Study of Parents and Children.^19^ The potential impact on inequalities in secondary school achievement of scaling up early years interventions is unknown, including what would be the likely impact of differences in implementation, such as rolling out universal or targeted interventions. The exposure variable was a multidimensional index of socioeconomic circumstances, derived using a latent class analysis of measures of parental education, social class, home ownership, household crowding, employment status and financial difficulties, all recorded during pregnancy.

First, the CDE of socioeconomic circumstances on the outcome, controlling for observed levels of school readiness (mediator) was modelled using a marginal structural model (MSM, box 1), enabling adjustment for baseline and exposure-induced mediator-outcome confounding. The degree of observed inequality between highest and lowest socioeconomic groups was represented by RRs using the estimated probabilities of the outcome from the MSM. Second, the mediator value was manipulated by shifting the distribution up or down (reflecting expected changes of an intervention, based on evidence from systematic reviews of randomised control trials). Inequalities were recalculated using the existing CDE model, only under the conditions of the new mediator values. Measures of both a ‘population yield’ (expected population prevalence of the outcome) and an ‘equity yield’ (inequality across socioeconomic groups) were generated from these models. Different ways of rolling out the intervention were examined. For example, in a scenario simulating a progressive universal intervention (where those in greatest need received a more intensive intervention), the mediator (school entry score) was shifted up by 0.8 SD for children from a low socioeconomic position and 0.2 SD for other children.

The modelled hypothetical interventions varied in impact, with inequalities remaining in every intervention scenario. The most successful scenarios provided more intensive intervention for disadvantaged children, with the progressive universal intervention estimated to reduce prevalence of poor educational achievement by 5% and inequalities by 10%. Overall, these analyses indicate that early years’ interventions on school readiness have only limited impact, but those delivered with more intensity for disadvantaged groups have the greatest potential to reduce socioeconomic inequalities in later school examination performance (all other things being equal, including intervention acceptability and effectiveness across social groups).

#### Limitations

In addition to standard assumptions and those raised in previous questions, users of this approach need to be cognisant of extrapolation of effects of the manipulated mediator outside of a range supported by the observed data (positivity assumption).^23^ By modelling hypothetical scenarios that vary in terms of eligibility, effectiveness and uptake, this method explicitly speaks the language of real-world interventions, but as with any method of assessing causal effects, including RCTs, there are many practical factors that affect the success of real-world implementation of interventions.

### Estimands for inequalities research in a mediation framework

Most mediation methods estimate the causal effect of the exposure. This necessitates: (1) that the exposure is well-defined in terms of a hypothetical intervention which changes the exposure and leads to the causal effect; (2) that the intervention must be directly linked to levels of exposure in the data. Together, these two requirements are known as the ‘consistency assumption’.^15^ For many exposures in the context of inequalities research, such as ‘socioeconomic status’, this assumption does not hold due to their complex, multifactorial nature. However, inequalities researchers often do not intend to intervene on or manipulate the exposure. Indeed, while policies can change some population socioeconomic circumstances, such as income,^24^ it is often unrealistic to intervene directly on the exposure, such as when considering ethnic differences in health.^25^ Rather, the intention is to estimate how intervening on the mediator would affect the magnitude of observed/descriptive health inequalities. This is arguably more informative for policymakers and relaxes the assumptions of consistency and non-confounding. Some approaches that implement these ideas have been proposed, with accessible examples of their application emerging.^24 26^

## Conclusions

This article outlines methods to address real-world policy questions about child health inequalities. What would be the inequalities impact of action on a wide range of known modifiable mediating pathways, and how much of the inequality in an outcome remains unexplained? If there are major mediators or key time points on a mediating pathway, how do we identify the most important ones? For a given mediating pathway, what would be the impact of a hypothetical intervention when implemented under different scenarios?

In addressing these questions, we have discussed approaches used to formally explore the mediating pathways between childhood socioeconomic circumstances and outcomes.

The methods outlined, using counterfactual based causal inference methodology, have the same assumptions as traditional mediation approaches (confounding, selection and information bias, consistency and positivity), but these assumptions are made explicit and are ideally examined through various types of sensitivity analysis.^14^ Simply using causal methods does not mean causal effects are quantified accurately—this depends on the accuracy of the DAG informing the analysis, the quality of measured variables and the extent of unmeasured confounding.^25^ Therefore, it is crucial that the development of a robust DAG incorporates expert knowledge and lived experience. The contribution of the causal inference approach lies in assessing the likelihood of differences between the statistical estimate and the target causal estimand.^27^

Future methodological developments may overcome some of the limitations outlined, and mediation techniques will become more accessible as they are implemented in standard statistical software packages. Nevertheless, understanding how best to communicate the messages from these analyses for policymakers and practitioners remains a crucial concern. In terms of data availability, population level linkage opens up the prospect of rich longitudinal data on multiple mediators over time,^17 28^ providing opportunities to better describe mediating pathways (such as identifying intervention opportunities, the role of critical periods and duration of effects) and for analysis of the cumulative mediating role of specific clusters of exposures.^29^

The motivation behind this paper is to support the population health research community to better understand the causal pathways through which health inequalities arise, to inform action to reduce and prevent them. Causal inference techniques continue to be refined and extended, but we hope that these illustrative examples provide encouragement for further use of causal mediation methods, in the effort to address important questions about the role of mediating factors in the generation and potential reduction of health inequalities.

## Data Availability

Data sharing not applicable as no datasets generated and/or analysed for this study.
